# Targeting the Hippo/YAP Pathway: A Promising Approach for Cancer Therapy and Beyond

**DOI:** 10.1002/mco2.70338

**Published:** 2025-08-29

**Authors:** Rajni Bala, Reecha Madaan, Onkar Bedi, Amrinder Singh, Ayushi Taneja, Renu Dwivedi, Gabriela Figueroa‐González, Octavio Daniel Reyes‐Hernández, Laura Itzel Quintas‐Granados, Hernán Cortés, Dietrich Büsselberg, Gerardo Leyva‐Gómez, Javad Sharifi‐Rad, William C. Cho

**Affiliations:** ^1^ University School of Pharmaceutical Sciences, Rayat Bahra University Kharar Punjab India; ^2^ Adesh College of Pharmacy Kurukshetra Haryana India; ^3^ Chitkara College of Pharmacy Chitkara University Rajpura Punjab India; ^4^ School of Pharmaceutical Sciences Bahra University Waknaghat Himachal Pradesh India; ^5^ Laboratorio de Farmacogenética UMIEZ Facultad de Estudios Superiores Zaragoza Universidad Nacional Autónoma de México Ciudad de México Mexico; ^6^ Colegio de Ciencias y Humanidades Plantel Cuautepec Universidad Autónoma de la Ciudad de México Ciudad de México Mexico; ^7^ Laboratorio de Medicina Genómica Departamento de Genómica Instituto Nacional de Rehabilitación Luis Guillermo Ibarra Ibarra Ciudad de México Mexico; ^8^ Weill Cornell Medicine‐Qatar, Education City, Qatar Foundation Doha Qatar; ^9^ Departamento de Farmacia Facultad de Química Universidad Nacional Autónoma de México Ciudad de México Mexico; ^10^ Universidad Espíritu Santo Samborondón Ecuador; ^11^ Centro de Estudios Tecnológicos y Universitarios del Golfo Veracruz Mexico; ^12^ Department of Medicine College of Medicine Korea University Seoul Republic of Korea; ^13^ Department of Clinical Oncology Queen Elizabeth Hospital Kowloon Hong Kong SAR China

**Keywords:** cancer, Hippo/Yap pathway, natural products, regulation, targets

## Abstract

Cancer represents a growing cause of death and a threat to public health worldwide; thus, there is an urgent need to understand its pathological mechanism and design effective therapies. The Hippo pathway regulates diverse cellular processes under physiological conditions; however, its dysregulation is associated with several types of cancer, including lung, pancreatic, colorectal, breast, and prostate cancer. Consequently, compounds targeting deregulated Hippo components represent potential treatments for a broad spectrum of cancers. Nonetheless, currently, there is limited information integrating the growing evidence of this potential. Therefore, the review's objective is to provide insight into the potential efficacy of targeting the Hippo/yes‐associated protein (YAP) pathway for cancer therapy. First, we describe the molecular mechanisms of the Hippo signaling pathway in physiological conditions and several cancer types. We then provide an overview of natural products and synthetic compounds targeting this pathway, highlighting their potential applications in treating diverse cancers. We also discuss relevant preclinical and clinical studies of compounds targeting the Hippo pathway in cancer. Finally, we summarize our findings and offer recommendations for future research. This review emphasizes the role of the Hippo/YAP pathway in cancer and the potential of natural products and synthetic compounds targeting this pathway for cancer treatment.

## Graphical abstract

The potential of natural compounds to modulate the Hippo/YAP signaling pathway, a critical regulator of cell proliferation, differentiation, and survival, is explored. Dysregulation of this pathway is associated with various cancers, making it a target for therapeutic intervention. The molecular mechanisms by which these natural products influence the Hippo/YAP pathway are highlighted, potentially leading to reduced tumor growth, apoptosis induction, and improved treatment outcomes when used in combination with conventional cancer therapies. Emphasis is placed on translational challenges and the need for clinical validation to optimize these natural compounds for therapeutic use.

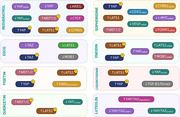

## 1 Introduction

Cancer is a leading cause of death globally, with an approximate annual death toll of 10 million people. The process of carcinogenesis involves multiple stages that work together and are closely linked. This process generates malignant cells that can proliferate and spread uncontrollably [1]. In multicellular organisms, the precise regulation of cell numbers during physiological processes, such as development and tissue regeneration, is crucial. Any dysregulation of this homeostatic control can contribute to the formation of cancer and tumors [2]. The global threat of tumors to human health is significant, requiring a deeper understanding of the underlying mechanisms. Cancerous cells undergo dynamic changes in their microenvironment and nutritional availability throughout tumor development [3]. Therefore, for cancer cells to persist, adapt, proliferate, and even alter their behavior to the extent of invasion and metastasis, they must adjust their metabolic and physiological processes in response to these changes [4]. One critical factor in tumor progression is the overactivation of signaling pathways associated with cancer and proliferative behaviors [5]. The key mechanism for controlling cell proliferation and survival is mediated by signaling pathways that transmit extracellular and intracellular stimuli to gene transcription [6]. Among these signaling routes is the Hippo pathway, a tumor suppressor pathway that regulates organ size, prevents apoptosis, and inhibits excessive cell proliferation [7].

The Hippo signaling pathway was initially identified as a critical regulator of organ size in Drosophila melanogaster. This discovery provided critical insights into the conservation of signaling mechanisms across species and included the recognition of four tumor suppressors: Warts (Wts), a nuclear dbf2‐related (NDR) family protein kinase; Salvador (Sav), a WW domain‐containing scaffold protein; Hippo (Hpo), a Ste20‐like protein kinase; and Mob‐as‐tumor suppressor (Mats), an adaptor protein. Additionally, the transcriptional co‐activator Yorkie (Yki) plays a crucial role in this pathway [8]. In mammals, this pathway has conserved functions with orthologous components. The mammalian orthologs of these proteins are yes‐associated protein (YAP)/transcriptional co‐activator with PDZ binding motif (TAZ), salvador homolog 1 (SAV1), large tumor suppressor kinase 1/2 (LATS1/2), and mammalian sterile 20‐like 1/2 (MST1/2) [9]. In contrast to other developmental pathways, Hippo activation typically signifies the completion of the developing process, whereas Hippo inactivation results in the induction of developmental and tumor‐related characteristics such as cell migration and proliferation. Consequently, Hippo is considered a tumor‐suppressive route [10]. Activation of the Hippo pathway is caused by several phosphorylation events [11]. MST1/2 (Hpo) is phosphorylated in response to upstream signals, and this phosphorylation then proceeds to phosphorylate MOB1A/B (Mats) and SAV1 (Sav), which helps to recruit LATS1/2 (Wts). On the other hand, when MST1/2 (Hpo), SAV1 (Sav), LATS1/2 (Wts), and MOB1A/B (Mats) are inactivated or degraded, YAP/TAZ (Yki) is nuclear localized. YAP/TAZ then interacts with VGLL4 (Tgi) to bind TEAD (Sd), leading to the upregulation of pro‐cancerous genes. The Hippo pathway has evolved to include additional components that influence LATS1/2, MST1/2, or YAP/TAZ activity [12]. The upregulation of YAP/TAZ, resulting from the Hippo pathway dysregulation, is a prevalent characteristic observed in numerous types of malignancies, such as non‐small‐cell lung cancer (NSCLC), glioma, pancreatic cancer, sarcoma, colorectal cancer, breast cancer, melanoma, and prostate cancer [13]. This overactivity of YAP/TAZ promotes the transcription of genes related to drug resistance, metabolic reprogramming, cancer survival, proliferation, invasion, migration, and immunosuppression [14]. Loss of Hippo pathway tumor suppressors or hyperactivation of YAP/TAZ can lead to tumor cell resistance to anticancer treatments, such as 5‐fluorouracil, paclitaxel, cisplatin, and doxorubicin. Drug sensitivity is restored by pharmacologically depleting or inhibiting YAP/TAZ or by re‐expressing tumor suppressors in the Hippo pathway. This suggests that target YAP/TAZ activity could combat drug resistance in cancer cells [8]. In light of the correlation between heightened YAP/TAZ levels and various types of cancer, therapeutic approaches aimed at combating cancer by targeting the Hippo pathway focus on directly or indirectly impeding the actions and roles of YAP and TAZ [15].

Numerous strategies targeting specific components of the Hippo pathway have been examined, including the development of medications that inhibit the activation of MST and LATS, the YAP/TAZ–TEAD interaction, or the palmitoylation pocket of TEAD [16]. Several of these approaches have demonstrated therapeutic benefits. For instance, verteporfin suppresses retinoblastoma by interfering with YAP–TEAD interactions [17]. Similarly, vinyl sulfonamide compounds attach to the palmitate‐binding pocket of TEAD, affecting the interaction of TEAD with YAP [18]. It is feasible to indirectly inhibit YAP/TAZ by focusing on their upstream stimulators. For example, erlotinib, an EGFR inhibitor, can interfere with YAP/TAZ activities, making it a potential option to treat malignancies triggered by the EGFR‐YAP/TAZ axis [19]. Interestingly, signaling through G protein‐coupled receptors (GPCRs) also plays a role in regulating YAP/TAZ. Inhibiting Gαq/11 with losartan [20] or stimulating Gαs with dihydrexidine [21] seems to boost the phosphorylation of YAP, leading to its subsequent degradation.

On the other hand, some naturally occurring phytochemicals derived from plants exhibit surprising anticancer action by modifying the Hippo pathway [22]. Numerous studies have shown that flavonoids (such as luteolin, naringin, fisetin, quercetin, and liquiritigenin) and stilbenoids (such as resveratrol) can cause cancer cells to undergo apoptosis by preventing their growth, migration, and differentiation. Flavonoids and stilbenoids raise the phosphorylated state of upstream components of YAP, namely, MST1/2 and LATS1, in the context of the Hippo signaling pathway. As a result, phosphorylation and nuclear translocation of TAZ and YAP are reduced. Furthermore, a recent study has shown that flavonoids may bind to the YAP–TEAD interaction interface and disrupt this complex [23]. Liquiritigenin, extracted from licorice, stimulates LATS to cause phosphorylation of YAP, inhibit tumor cell proliferation, and serve as a hepatoprotective agent [24]. Similarly, decursin (extracted from the Korean Dang Gui root) can phosphorylate LATS1 and YAP and reduce YAP production at the protein level (but not mRNA) [25]. Thus, decursin prevents HepG2 liver cancer cells from proliferating by inducing apoptosis, arresting the cell cycle, and reducing cell division. Alkaloids are another category of naturally occurring chemicals that exhibit promising anticancer properties and have been explored as potential anticancer medications [26]. Specific Hippo signaling proteins are expressed and phosphorylated in part by alkaloids. For instance, the alkaloid matrine, found in Sophora flavescens Ait, inhibits the survival of tumor cells by activating mitochondrial division of MIEF1 via the LATS2‐Hippo pathway [27]. Another alkaloid, narciclasine, has also been investigated for its potential to inhibit the proliferation of mesothelioma cells that produce high levels of YAP protein. Narciclasine competes with TEAD4 to interact with YAP, thereby inhibiting tumor growth [28]. Furthermore, natural naphthoquinone shikonin, extracted from traditional Chinese medicine, also demonstrated a potent ability to suppress the growth of cancer cells by phosphorylating AMPK [29]. Finally, CL‐6, a recently identified curcumin derivative, exhibits anticancer effects by killing GC cells via the expression of Hippo signaling [30].

The efficacy of compounds specifically targeting Hippo pathway‐dependent tumor cell proliferation demonstrates a clear potential for advancing cancer therapy. Nonetheless, currently, there is limited information integrating the growing evidence of this potential. Therefore, the review's objective is to provide insight into the potential efficacy of targeting the Hippo/YAP pathway for cancer therapy. First, we describe in detail the molecular mechanisms of the Hippo signaling pathway in physiological conditions and several cancer types, including experimental models. We then provide an overview of natural products and synthetic compounds targeting the Hippo/YAP pathway, with potential applications for treating diverse cancers. We also discuss relevant preclinical and clinical studies of these compounds targeting the Hippo pathway in cancer, supporting the notion that this pathway represents a promising opportunity for cancer treatment. Finally, we summarize our findings and offer recommendations for future research. This review highlights the significance of the Hippo/YAP pathway in cancer and explores the potential of natural products and synthetic compounds that target this pathway for cancer treatment.

## 2 The Hippo/YAP Pathway in Cancer

The Hippo pathway has important functions in organisms; however, emerging evidence suggests that it is also a critical regulator of the initiation and progression of several cancer types. Thus, its study has gained relevance in recent years. A main feature of the Hippo pathway is its ability to modulate the activity of YAP/TAZ. For example, under normal physiological conditions, this pathway prevents YAP/TAZ from entering the nucleus, thereby inhibiting their interaction with transcription factors that promote the expression of oncogenes. However, when the Hippo pathway is disturbed, unrestrained YAP/TAZ activity can lead to increased cell proliferation and survival, contributing to cancer development and progression. These mechanisms are discussed in detail below.

**FIGURE 1:**
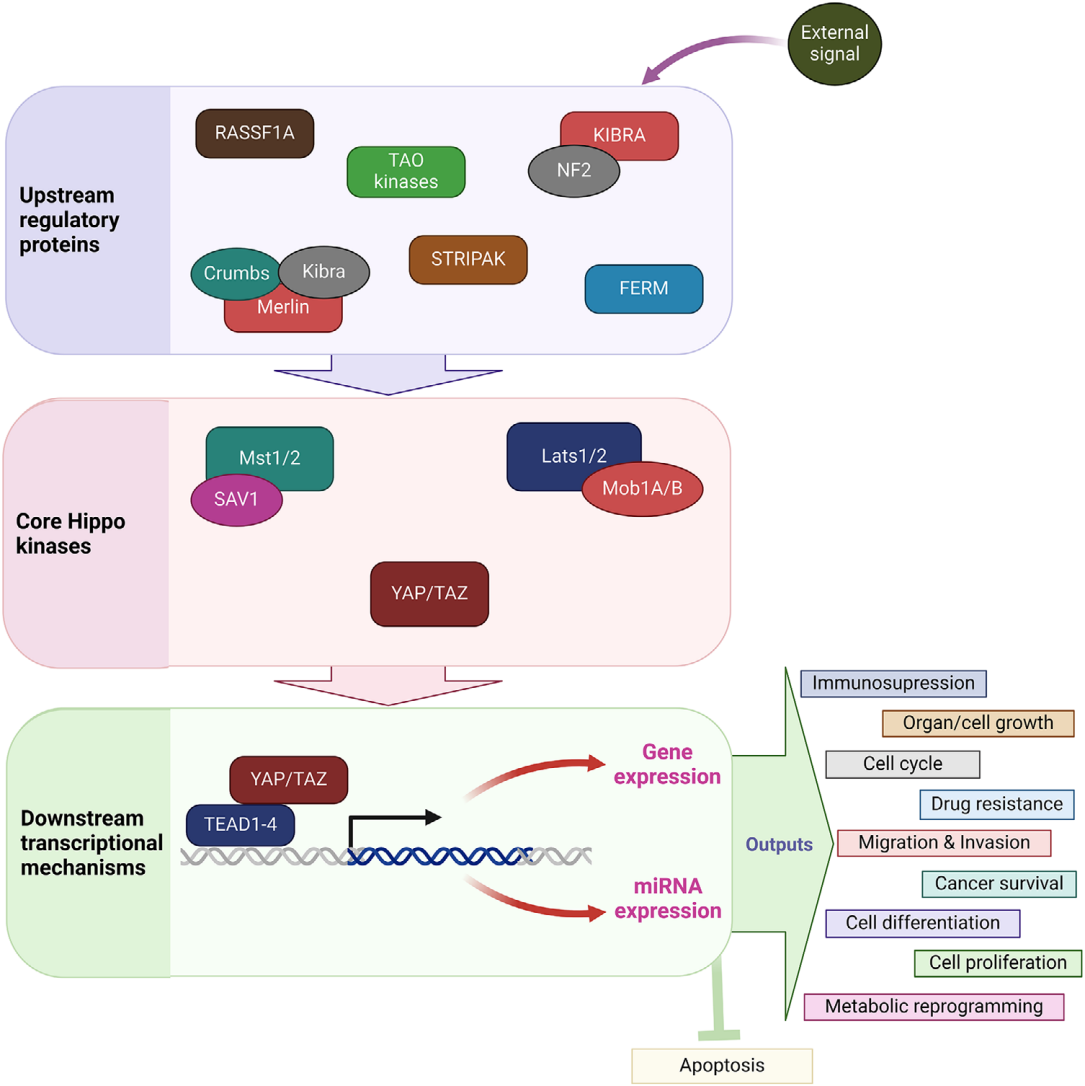
Principal components of the Hippo pathway. This pathway can be divided into three main components: upstream regulatory proteins, central core kinases, and downstream transcriptional mechanisms. Extracellular signals activate the upstream regulatory factors, transmitting these signals to the core kinases. These signals are subsequently passed on to downstream effectors, leading to the expression of genes and microRNAs. LATS1/2, large tumor suppressor kinase 1/2; MST1/2, mammalian sterile 20‐like 1/2; SAV1, salvador homolog 1; YAP, yes‐associated protein. *Source*: Created in https://BioRender.com.

The Hippo signaling pathway regulates cell growth, proliferation, apoptosis, differentiation, and organ size (Figure 1); it is evolutionarily conserved and functional across many organisms, from Drosophila to mammals. This pathway comprises approximately 30 proteins and can be categorized into three principal components: upstream regulatory proteins, core kinases at the center, and downstream transcriptional mechanisms. The initiation of the Hippo signaling pathway involves the reception of growth inhibition signals from the extracellular environment by upstream regulatory proteins. These signals are then relayed to the core kinases before reaching downstream effectors. Recent research has shed light on the involvement of the Hippo signaling pathway in cancer development, which acts as a tumor suppressor mechanism [31, 32, 33]. The core kinase cassette in Drosophila's Hippo pathway includes four essential proteins: Wts, Sav, Hpo, and Mats. Wts is a protein kinase belonging to the NDR family; mutations in this protein lead to abnormal tissue growth in various epithelial tissues in Drosophila [34]. Mutations in Sav lead to increased cell numbers due to enhanced cell proliferation and reduced cell death [35]. Loss of Hpo leads to tissue overgrowth similar to what is observed in Wts and Sav mutants. Hpo binds to Sav and phosphorylates it; this interaction promotes Wts phosphorylation [36]. Lastly, Mats associates with Wts and acts as an activating subunit for Wts. Mutations in Mats trigger excessive cell proliferation and tumor formation [37]. Yki serves as the downstream target that undergoes phosphorylation and subsequent inactivation by Wts. Additionally, an increase in Yki expression leads to excessive tissue growth. Upstream regulators Merlin and Expanded, membrane‐associated F for protein 4.1, E for ezrin, R for radixin, and M for moesin (FERM) (4.1/ezrin/radixin/moesin) domain‐containing proteins, are essential for halting proliferation and inducing apoptosis in developing imaginal discs [38]. When active, Yki forms a complex with the transcription factor scalloped (Sd), translocating it to the nucleus where it promotes the transcription of target genes involved in vital processes such as organ growth, cell cycle advancement, and apoptosis inhibition through factors like cyclin E and DIAP1 [39]. Moreover, Yki triggers the expression of *bantam* microRNA, which plays a critical role in promoting cell proliferation and suppressing apoptosis [40].

### 2.1 YAP/TAZ Phosphorylation: Key for Pathway Regulation

**FIGURE 2:**
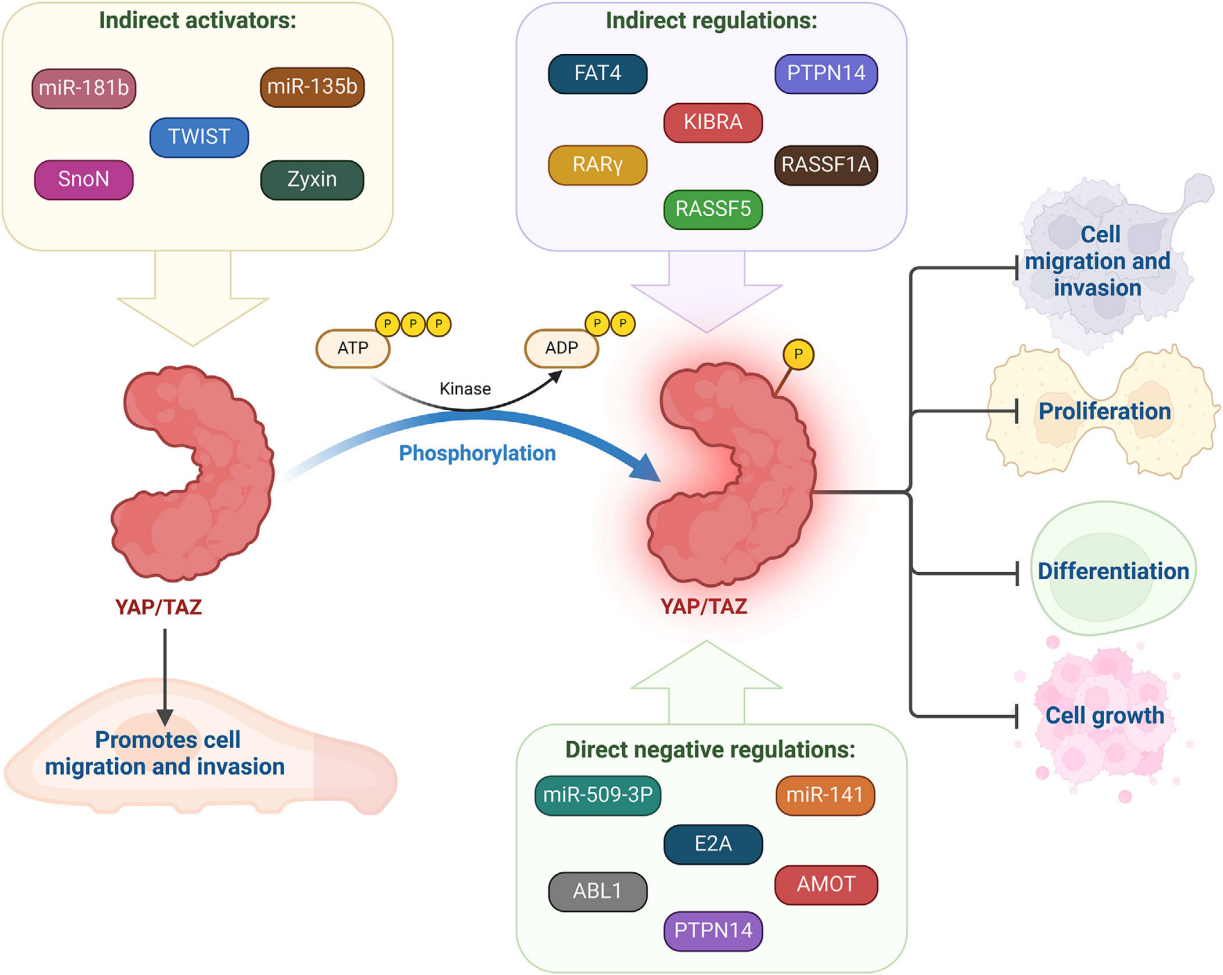
YAP/TAZ phosphorylation and its implications with cancer. In addition to LATS1, several direct negative regulators of YAP and TAZ, including miR‐509‐3p, miR‐141, E2A, *ABL1*, AMOT, and PTPN14, as well as indirect regulators like FAT atypical cadherin 4 (FAT4), KIBRA, Ras association domain family 5 (RASSF5), RASSF1A, PTPN14, and retinoic acid receptor γ (RARγ), decrease cell migration and invasion. Migration and/or invasion are facilitated by indirect activators of YAP and TAZ, such as miR‐181b, miR‐135b, TWIST, SnoN, and Zyxin. YAP, yes‐associated protein. *Source*: Created in https://BioRender.com.

The Hippo signaling pathway in mammals primarily consists of MST1/2, LATS1/2, MOB1A/B, YAP, and its homolog TAZ, also known as the WW domain containing transcription regulator 1 (WWTR1) [41]. The upstream kinases of this pathway, MST1 and MST2, collaborate with the adaptor protein SAV1/WW45 to phosphorylate and activate LATS1 and LATS2. Subsequently, the activated LATS kinases, in conjunction with MOB1A/B, phosphorylate the YAP and TAZ effector proteins. This phosphorylation event leads to the exclusion of YAP/TAZ from the nucleus by 14‐3‐3 proteins, ultimately resulting in their degradation in the cytoplasm [42] (Figure 2). The kinase module (MST1/2 and LATS1/2) functions by phosphorylating and inactivating the members of the transcriptional module, YAP (Yorkie or Yki), and its paralogue TAZ (encoded by the *YAP1* and *WWTR1* genes, respectively), which were identified as oncogenes soon after their discovery [43]. Inactive and phosphorylated YAP/TAZ are held in the cell's cytoplasm before being broken down. Nuclear YAP/TAZ binds to the TEAD family of transcription factors, which includes TEAD1‐4 (scalloped or Sd), to activate the expression of gene programs that promote proliferative and survival‐enhancing processes when there is not an active kinase cascade [44].

### 2.2 Regulation of the Hippo/YAP Pathway

Cancer cells receive various signals that can regulate Hippo signaling, playing a crucial role in tumor development. For example, activating GPCRs associated with Gas leads to LATS phosphorylation and subsequent inactivation of YAP. Conversely, there is a correlation between the activation of YAP and the inhibition of LATS when Gα12/13‐coupled GPCRs are activated [21]. In addition to GPCRs, membrane receptor tyrosine kinases such as EGFR and ERBB4 influence YAP production and proliferation. Activation of EGFR stimulates PI3K and PDK1 pathways, connecting them to the Hippo pathway via the adaptor protein SAV1/WW45. Activation of PDK1 leads to the dissociation of Hippo core kinases from the scaffolding protein SAV1/WW45, which, in turn, causes the expression of YAP target genes, dephosphorylation, and nuclear translocation of YAP, and inactivation of LATS [45].

In addition to receptor‐mediated control, the Hippo signaling pathway is also regulated by “mechanical” inputs from both the cell surface and the inside of the cell. Mechanical cues that bind to intercellular junctions (such as tight and adherence junctions) and cytoskeleton elements (including actomyosin, microtubules, F‐actin, and perhaps centrosomes) regulate Hippo signaling [46]. Mechanical regulation of the Hippo pathway is an essential aspect of how cells respond to their physical environment. Mechanical signals, such as extracellular matrix (ECM) stiffness, cell shape, actomyosin contractility, and mechanical stretching, regulate YAP/TAZ activity, thereby influencing cell growth, differentiation, and organ size. In breast cancer, cells grown on stiff matrices (such as those mimicking tumor stiffness) exhibit nuclear localization of YAP/TAZ, promoting cell proliferation and survival. In contrast, when cells are cultured on soft matrices (mimicking normal breast tissue), YAP/TAZ are retained in the cytoplasm, reducing cell proliferation. The stiffness of the ECM modulates actomyosin contractility through integrins, leading to Rho GTPase activation and cytoskeletal tension, which drives YAP/TAZ nuclear localization [46]. On the other hand, when cells spread over a larger surface area, they adopt a flatter morphology, which increases mechanical stress and actin tension. This leads to nuclear localization of YAP/TAZ, driving cell growth and survival. Conversely, cells constrained to small, rounded shapes exhibit cytoplasmic retention of YAP/TAZ, resulting in reduced proliferation. The regulation of YAP/TAZ by cell shape is controlled by actin cytoskeleton tension and focal adhesions, which sense and transmit mechanical signals into the Hippo pathway [47].

Mechanical stretching (mimicking breathing) induces YAP/TAZ activation and nuclear localization in lung epithelial cells, leading to upregulation of proliferation‐related genes. Cells in regions subjected to mechanical strain respond by promoting YAP/TAZ‐driven tissue repair and growth. Cyclic stretching induces cytoskeletal reorganization and focal adhesion signaling, activating YAP/TAZ through actin polymerization and mechanical stress [48]. In endothelial cells, the inhibition of actomyosin contractility using inhibitors such as blebbistatin (a myosin II inhibitor) prevents the nuclear localization of YAP/TAZ, thereby decreasing cell migration and proliferation. On the other hand, increased actomyosin contractility promotes the nuclear localization and activity of YAP/TAZ. Actomyosin contractility generates intracellular tension, which promotes YAP/TAZ activity by disrupting the Hippo kinase complex, reducing the phosphorylation of YAP/TAZ, and allowing it to enter the nucleus [49]. On the other hand, when epithelial cells grow at high density, YAP/TAZ is sequestered in the cytoplasm due to contact inhibition. In this process, cell–cell junctions signal to the Hippo pathway to inactivate YAP/TAZ. This suppresses cell proliferation and prevents overgrowth. YAP/TAZ enters the nucleus when cell density is low and promotes proliferation. E‐cadherin‐mediated cell–cell adhesion activates components of the Hippo pathway, such as LATS1/2, which phosphorylate and inactivate YAP/TAZ, keeping them in the cytoplasm [2]. Mechanosensitive ion channels, such as Piezo1, are activated by mechanical stretching in endothelial cells. This activation leads to an influx of calcium, which influences cytoskeletal tension and ultimately regulates YAP/TAZ activity. Mechanical stimuli in endothelial cells thus control YAP/TAZ localization and their role in angiogenesis and vascular homeostasis. Piezo1 activation in response to mechanical force triggers calcium signaling, which influences cytoskeletal dynamics and impacts YAP/TAZ activity, ultimately driving cellular responses to mechanical stress [50]. Furthermore, dysregulated cytoskeleton components are frequently observed in cancer cells, which may also contribute to the activation of YAP/TAZ during carcinogenesis. The apical membrane protein neurofibromatosis 2 (NF2/Merlin) is a well‐known tumor suppressor inactivated in many malignancies and is one of the most studied upstream regulators of the Hippo signaling pathway. Merlin attracts and binds the LATS kinases to the plasma membrane, facilitating MST1/2 and SAV1‐mediated phosphorylation and activation [51]. Additionally, phosphorylated Merlin directly regulates LATS activity by blocking the tumor‐promoting actions of the E3 ubiquitin ligase CRL4‐DCAF1 in the nucleus [52]. LATS kinases are the primary regulators of YAP/TAZ. The transcriptional activity of YAP and TAZ is negatively regulated by LATS1/2 by phosphorylation at one or more serine residues. Besides LATS1/2, AKT and JNK proteins also mediate YAP serine phosphorylation. Furthermore, YES, Src, or c‐ABL kinases contribute to tyrosine phosphorylation, adding another level of YAP regulation [53].

### 2.3 Mutations, Cellular Localization, and Dysregulation in the Hippo/YAP Pathway

The Hippo pathway regulates tissue growth and controls programmed cell proliferation. Dysregulation of this system has been implicated in various human malignancies, including breast, lung, colon, and liver cancers [54]. Even though the Hippo signaling pathway is often dysregulated across various cancer types, mutations within the pathway are relatively rare, typically present in <10% of cancer cases [55]. The Hippo pathway's dysregulated key components include NF2, MST, LATS, and YAP/TAZ. However, the *NF2* gene is the only Hippo pathway element found to mutate in cancer and become inactive. *NF2* mutations, which result in the formation of benign nervous system tumors, are the cause of Type 2 neurofibromatosis [56]. Disruption of this system results in hypermethylation of the *MST* promoter. Additionally, protein 1A of the RAS association domain family (RASSF1A) can positively influence MST [57]. In humans, the *RASSF1A* gene is frequently deleted or methylated in cancers of the breast, stomach, nasopharynx, liver, lung, pancreas, prostate, and skin [58]. Reports also indicate *LATS* gene hypermethylation in human malignancies [59]. Furthermore, functional mutations in the *LATS2* gene can render it inactive, affecting approximately 40% of mesothelioma cases [60].

**FIGURE 3:**
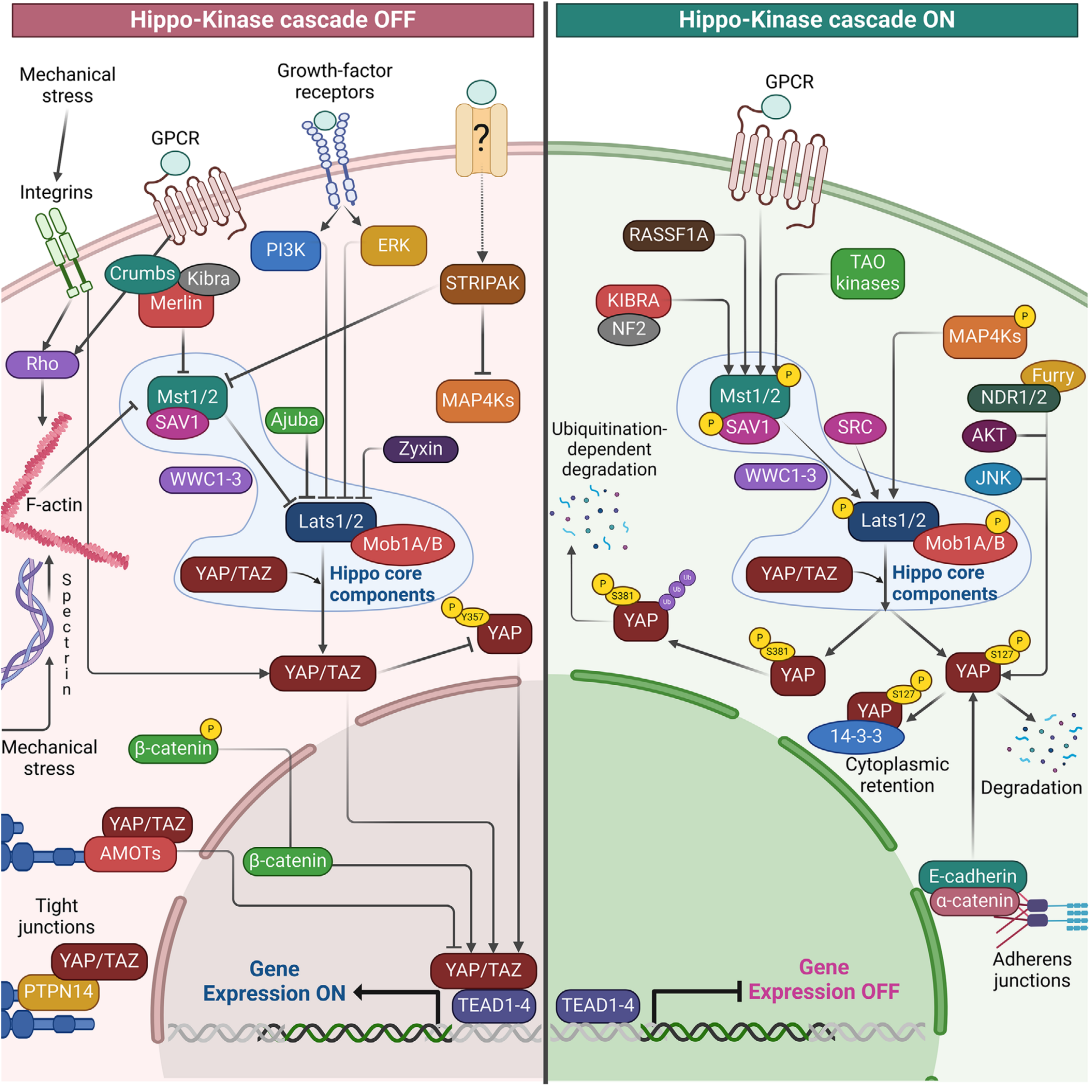
The Hippo–YAP pathway signaling. The regulation of the Hippo pathway‐YAP/TAZ in mammalian cells involves a multitude of signaling mechanisms, including cell polarity and cell adhesion proteins (α‐Catenin, PTPN14, AMOT), mechanical signals (ECM stiffness, cell contact, and geometry), and signal transduction pathways (GPCR, growth‐factor receptors, integrins, Src, MAP4Ks, TAO kinases, STRIPAK complex). The core components of the Hippo–YAP pathway are the kinases MST1/2 and LATS1/2, along with their cofactors salvador (SAV) and scaffolding proteins MOB1A/B, transcription co‐activators YAP and TAZ, and the TEAD1–TEAD4 family of transcription factors. When the Hippo–YAP pathway is active (ON), MST1/2 phosphorylates LATS1/2, phosphorylating and degrading YAP and TAZ. When the pathway is inactive (OFF), YAP/TAZ dephosphorylates accumulates in the nucleus and binds with TEADs to induce gene transcription, promoting the transcription of genes like CTGF and CYR61. Other molecules, such as NDR1/2, Src, NLK, AMPK, and JNK, directly phosphorylate and regulate YAP/TAZ, alongside kinase‐independent mechanisms. Various upstream mediators, such as adhesion proteins, mechanotransduction, and other signaling pathways, regulate YAP activity. LATS1/2, large tumor suppressor kinase 1/2; MST1/2, mammalian sterile 20‐like 1/2; YAP, yes‐associated protein. *Source*: Created in https://BioRender.com.

Unsurprisingly, YAP acts as an oncogene due to its pivotal role as the primary downstream effector of the Hippo pathway (Figure 3). Dysregulation of this system leads to elevated YAP/TAZ expression and nuclear localization in various human cancer types. Activated YAP/TAZ in cancer cells contributes to aggressive behavior, stem cell‐like properties, and resistance to chemotherapy. Studies using transgenic mouse models of liver cancer have provided a comprehensive understanding of the carcinogenic role of YAP/TAZ [61]. For example, a study demonstrated that the tissue‐specific deletion of MST1/2 and SAV1 is sufficient to initiate liver carcinogenesis. Hippo‐regulated genes implicated in immunological and inflammatory responses are enriched in transcriptional profiles [62].

**FIGURE 4:**
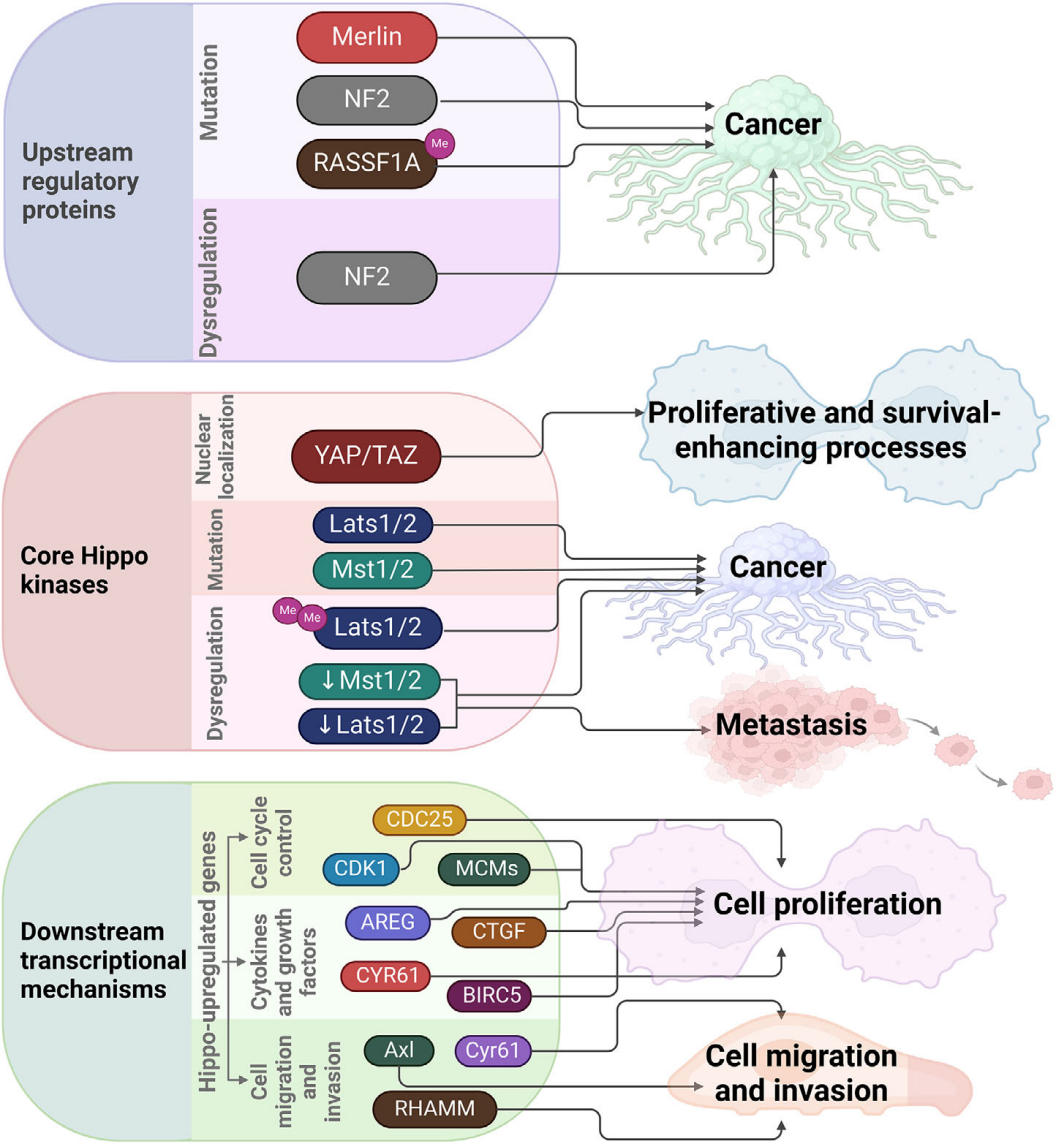
Relationship between Hippo signaling pathway and cancer. Dysregulation and/or mutations in the upstream regulatory proteins of the Hippo pathway are associated with cancer development. Mutations or dysregulation of core Hippo kinases are linked to cancer and metastasis, respectively. The nuclear localization of YAP/TAZ is associated with proliferative and survival‐enhancing processes—upregulation of Hippo‐regulated genes in cancer results in increased cell proliferation, migration, and invasion. LATS1/2, large tumor suppressor kinase 1/2; MST1/2, mammalian sterile 20‐like 1/2; YAP, yes‐associated protein. *Source*: Created in https://BioRender.com.

YAP/TAZ has a variety of roles in the cell that are necessary for tumor growth. For instance, by upregulating numerous genes involved in cell cycle control (such as *CCND1*, *CDK1*, *CDC25*, and *MCMs*), YAP/TAZ encourages cell proliferation [63]. Additionally, it stimulates anchorage‐independent proliferation in various cancer cells [64]. Similarly, mutations in the S127 or S381 residues enhance YAP's transforming activity [65]. Additionally, YAP activation promotes the production of cytokines and growth factors that can stimulate cell proliferation, including AREG, CTGF, cysteine‐rich angiogenic inducer 61 (CYR61), and BIRC5. YAP and TAZ also contribute to cell migration and invasion in a broad range of cancer cell types, including those from cholangiocarcinomas, colon cancer, endometrial cancer, gastric cancer, gliomas, HCC, mammary epithelium, neuroblastomas, OSCC, ovarian granulosa cell tumors, and pancreatic cancer [66]. Similar to LATS1, additional direct negative regulators of YAP and TAZ, such as miR‐509‐3p, miR‐141, E2A, ABL1, AMOT, and PTPN14, as well as indirect regulators, such as FAT atypical cadherin 4 (FAT4), KIBRA, RASSF5, RASSF1A, PTPN14, and retinoic acid receptor γ (RARγ), decrease cell migration and invasion [67]. Conversely, indirect activators of YAP/TAZ (e.g., miR‐181b, miR‐135b, TWIST, SnoN, and Zyxin) facilitate these cellular processes [68]. YAP/TAZ regulates cell motility through the mechanical limitation of focal adhesion and cytoskeletal maturation [69]. Furthermore, by upregulating specific downstream targets, including Axl, Cyr61, and the receptor for hyaluronan‐mediated motility (RHAMM), YAP/TAZ facilitates cell migration and invasion [70]. The retrospective examination of tumor specimens proves that disruption of the Hippo pathway promotes cancer spread (Figure 4). In‐line with their tumor‐suppressive roles, reduced expression or activity of MST1/2 and LATS1/2 leads to metastasis in a subset of cancers. For example, the decreased expression of LATS1/2 (usually due to promoter hypermethylation) correlates with advanced clinical stages in breast, cervical, colorectal, gastric, lung, ovarian, prostate, and kidney cancers. Specifically, it is associated with lymph node metastasis in breast, colorectal, gastric, and lung cancers [71]. Abnormally elevated YAP and TAZ activity within cancer cells facilitates metastasis, as demonstrated in xenograft mouse models [72]. Conversely, deactivating YAP/TAZ inhibits metastasis in various animal models [73].

In summary, the normal activity of the Hippo pathway is fundamental for maintaining cellular homeostasis and avoiding tumor formation. Conversely, the dysregulation of this pathway is associated with the development of several cancer types. Therefore, the Hippo pathway represents an exciting candidate for investigating novel therapeutic agents with anticancer actions.

### 2.4 Crosstalk Between the Hippo/YAP and Other Pathways

With its key effector YAP, the Hippo pathway is a central regulator of cell growth, organ size, and tumor suppression. The interaction between the Hippo/YAP pathway and other signaling pathways in cancer influences tumor progression, metastasis, drug resistance, and the tumor immune microenvironment. Understanding these interactions is essential for developing new therapeutic strategies.

The following Table 1 summarizes the therapeutic implications of Hippo/YAP crosstalk.

**TABLE 1:** Cancer therapeutic implications of Hippo/YAP crosstalk with other pathways.

Crosstalk target	Disease or functional outcome in cancer	Potential therapy focus/strategy or regulator	Effect on Hippo/YAP–Src axis or effect on tumor/immunity	Refs.
Src–YAP axis	NSCLC (non‐small cell lung cancer)	Src inhibitors	Reduce YAP activity, suppress tumor growth, and metastasis	[74, 75]
	Cancers	Combination therapies	Targeting both Src and YAP may improve outcomes	[74]
	Renal fibrosis	FXR agonists	Inhibit Src, enhance Hippo activity, protect against fibrosis	[76]
YAP/TAZ–SMAD2/3	Various	Modulating cell density, TGFβ pathway	—	[77]
ROR1–HER3–YAP	Breast (bone mets)	Targeting ROR1, HER3, lncRNAs	—	[78]
Hippo/YAP crosstalk with YAP/THBS1/FAK and YAP/HMGB1/FAK axes	Breast	Inhibiting focal adhesion, metastasis	—	[79, 80]
	Gastric cancer	HMGB1	Activation of FAK Downstream outcome: proliferation, migration	[79]
	Breast cancer	THBS1	Activation of FAK Downstream outcome: adhesion, invasiveness	[80]
Hippo/YAP crosstalk with TAZ/IL‐34 axis	TNBC	Targeting immune suppression		[81, 82]
	Target for cancer therapy	YAP/TAZ–TEAD	Drives tumor growth, immune suppression	[83]
	Potential target in TNBC	TAZ/IL‐34	Increases TAM infiltration, immunosuppression	[84]
	Needs careful therapeutic balance	Hippo pathway modulation	Impacts tissue regeneration and cancer risk	[77, 81, 82, 85, 86]
Hippo/YAP crosstalk with TGFβ pathway	Tumor suppression limits invasion	RASSF1A	Inhibitory effect on YAP/SMAD	[87]
	Proliferation, gene activation	ITCH	Promotes the YAP/SMAD complex	[88]
	Context‐dependent gene expression	Cell Density	Modulates localization of the YAP/SMAD complex	[77, 89]
Hippo/YAP crosstalk with KRAS	Drives proliferation, resistance	KRAS	Hippo/YAP and KRAS pathways	[90, 91]
	Essential for tumor progression	YAP/TAZ	Mediates KRAS effects, drug resistance	[90, 92]
	Aggressive, drug‐resistant subtypes	SFK	Modulates KRAS/YAP, promotes EMT	[93]
Hippo/YAP crosstalk with PI3K/Akt pathway	Endometrial, liver, oral cancer	YAP/TAZ inhibition	Reduces PI3K/Akt signaling, tumor growth	[94, 95, 96]
	Liver cancer, nephropathy	PI3K/Akt pathway inhibition	Attenuates disease progression	[95, 97]
	Hepatocellular carcinoma	Combined YAP and EGFR inhibition	Synergistic cytotoxicity in cancer cells	[98]
	Colon cancer	Natural compounds (DIM) + PI3K inhibitors	Enhanced anti‐proliferative effects	[99]

Abbreviations: FAK, focal adhesion kinase; NSCLC, non‐small‐cell lung cancer; TAM, tumor‐associated macrophage; TNBC, triple‐negative breast cancer; YAP, yes‐associated protein.

The Hippo/YAP and other pathways, such as Src, TGFβ, KRAS, Wnt/β‐catenin, and PI3K/Akt pathways, are deeply interconnected. Their crosstalk plays vital roles in development, tissue maintenance, and disease. Disruption of this balance can lead to cancer and other disorders, making the regulatory mechanisms between these pathways promising targets for future therapies.

#### 2.4.1 Src Pathway

The Hippo/YAP pathway and the Src kinase pathway are essential regulators of cell growth, survival, and cancer progression. Recent research highlights noteworthy crosstalk between these pathways, influencing tumor development, metastasis, drug resistance, and fibrosis. Understanding their interaction offers insights into potential therapeutic targets for cancer and other diseases.

Src kinase activates YAP through direct phosphorylation, repression of Hippo kinases, and Hippo‐independent mechanisms, promoting drug resistance and metastasis in non‐small cell lung cancer. Targeting the Src–YAP axis is a potential therapeutic approach [74].

Furthermore, Src phosphorylates and inhibits LATS1, a key kinase in the Hippo pathway, thereby reducing its tumor suppressor function. This leads to increased nuclear YAP activity and tumorigenesis [74, 75].

Additionally, YAP (and its Drosophila homolog Yki) can transcriptionally activate Src, which, in turn, further enhances YAP activity, creating a positive feedback loop that promotes tumor cell migration and metastasis [100].

In kidney disease, the activation of the nuclear receptor FXR inhibits Src, thereby enhancing Hippo pathway activity and promoting YAP cytosolic retention, which protects against renal fibrosis. Additionally, the inhibition of Src or activation of FXR decreases fibrosis markers and YAP target gene expression [76].

#### 2.4.2 TGFβ Pathway

Hippo and TGFβ signaling pathways are key regulators of cell growth, tissue homeostasis, and cancer progression. Recent research highlights extensive crosstalk between these pathways, particularly involving the Hippo effectors YAP/TAZ and the TGFβ mediators SMADs, which collectively influence gene expression, cell fate, and tumor behavior.

YAP/TAZ forms complexes with SMAD2/3 (TGFβ pathway) in a cell density‐dependent manner, primarily in the nucleus under sparse conditions, influencing gene expression and potentially contributing to cancer progression [77].

YAP/TAZ from the Hippo pathway physically interacts with SMAD2/3 from the TGFβ pathway, forming complexes that regulate gene transcription. TGFβ stimulation enhances the formation of these complexes, particularly under low cell density, and promotes their nuclear localization, which is crucial for gene regulation [77, 89, 101].

The extent and location of YAP/TAZ–SMAD2/3 complex formation depend on cell density and epithelial polarity. Sparse cultures favor nuclear complexes and active gene transcription, whereas dense cultures restrict complexes to the cytoplasm, reducing transcriptional activity [77, 89].

TGFβ signaling targets the Hippo scaffold protein RASSF1A for degradation through the E3 ubiquitin ligase ITCH. The loss of RASSF1A enables YAP to associate with SMAD2, facilitating their nuclear translocation and the activation of TGFβ target genes. Consequently, RASSF1A functions as a molecular switch, restraining the interaction between YAP and SMAD2 while limiting TGFβ‐induced invasion and gene expression [87, 88].

YAP can form distinct complexes with either SMADs, which promote proliferation, or p73, which promotes apoptosis. The balance between these complexes is dynamically regulated by upstream signals, with ITCH expression tipping the switch toward YAP–SMAD (proliferative) complexes [88].

Crosstalk can have opposing effects depending on the context. For example, the collaborative activation of CYR61 by YAP/TEAD4 and TGFβ/SMAD2/3 exerts a tumor‐suppressive effect in hepatocellular carcinoma, counteracting malignant transformation and growth [102].

Mathematical and experimental models indicate that YAP/TAZ can modulate TGFβ receptor activity and function as nuclear retention factors for SMADs, fine‐tuning the transcriptional response to TGFβ signals [88, 101].

#### 2.4.3 KRAS Pathway

The Hippo/YAP and KRAS pathways are central regulators of cell growth, survival, and cancer progression. The recent research has highlighted significant crosstalk between these pathways, influencing tumor development, metastasis, and resistance to targeted therapies, particularly in cancers such as pancreatic ductal adenocarcinoma (PDAC).

Some aggressive cancer subtypes, such as basal‐like PDAC, display reduced dependence on KRAS and heightened reliance on YAP/TAZ activity, often driven by Src family kinase (SFK) signaling [93]. YAP is crucial for the progression of KRAS‐mutant pancreatic neoplasia to invasive cancer. Deleting YAP stops tumor progression while sparing normal tissue, highlighting its specific role in KRAS‐driven cancers [90].

Oncogenic KRAS can activate YAP/TAZ, the main effectors of the Hippo pathway, through mechanisms like OTUB2‐mediated deubiquitination and SUMOylation, resulting in increased YAP/TAZ activity independent of Hippo pathway inactivation [91].

Moreover, YAP serves as a critical transcriptional switch downstream of KRAS‐MAPK signaling, enhancing the expression of genes that promote neoplastic proliferation and tumor progression [90, 103]. On the other hand, SFKs interact with both KRAS and YAP, modulating their activities and contributing to aggressive cancer phenotypes and drug resistance [93].

Activation of YAP/TAZ confers resistance to KRAS inhibitors by inhibiting pro‐apoptotic genes and activating survival pathways (e.g., SLC7A5/mTORC1 axis) [92]. While inhibiting YAP/TAZ or their interaction with TEAD transcription factors can overcome both intrinsic and acquired resistance to KRAS inhibitors, presenting a promising approach for precision oncology [92, 104].

Crosstalk between the Hippo/YAP and KRAS pathways is vital for cancer development, progression, and resistance to therapy. YAP/TAZ function as essential effectors downstream of KRAS, and their activation can circumvent KRAS dependency in some cancer subtypes. Targeting the Hippo–YAP/TAZ axis presents a promising approach to overcoming resistance to KRAS‐targeted therapies and enhancing outcomes in KRAS‐mutant cancers.

#### 2.4.4 Wnt/β‐Catenin Pathway

The Hippo/YAP and Wnt/β‐catenin pathways are key regulators of cell proliferation, differentiation, organ size, and tissue homeostasis. Increasing evidence indicates that these pathways do not function independently; instead, they engage in complex crosstalk that influences each other's activities in both normal physiology and disease, including cancer and neurodegeneration.

YAP/TAZ, effectors of the Hippo pathway, can bind to β‐catenin or disheveled, modulating Wnt/β‐catenin signaling depending on the cellular context. Phosphorylated YAP/TAZ can sequester β‐catenin in the cytoplasm, preventing its nuclear translocation and thereby inhibiting the expression of Wnt target genes. Conversely, when Hippo signaling is inactive, YAP/TAZ can enter the nucleus and cooperate with β‐catenin to drive transcriptional programs [105, 106, 107, 108].

YAP interacts with β‐catenin (Wnt pathway), acting as a transcriptional co‐regulator. Nuclear YAP upregulates β‐catenin, whereas cytoplasmic YAP suppresses it. This crosstalk is implicated in both fibrosis and cancer [105].

Both pathways use similar molecular machinery, including ubiquitin ligase complexes, and can affect each other's stability and localization of key proteins [109, 110].

Crosstalk between these pathways is crucial for proper tissue growth, organogenesis, and the maintenance of stem cells. In zebrafish embryos, Wnt/β‐catenin activity directly regulates YAP/TAZ activity, with both pathways showing overlapping expression in key developmental regions [106, 111, 112]. The interaction between Hippo/YAP and Wnt/β‐catenin is crucial for tissue repair and regeneration, ensuring a balance between cell proliferation and differentiation [106, 111, 112].

Dysregulation of either pathway or their crosstalk can drive tumorigenesis. In gastrointestinal and prostate cancers, YAP/TAZ and β‐catenin work together to promote cell proliferation and survival. In colorectal cancer, YAP/β‐catenin/TBX5 complexes are essential for tumor cell growth [107, 108, 109, 110, 113].

Both pathways and their interaction are implicated in diseases such as Huntington's, Alzheimer's, and ALS (amyotrophic lateral sclerosis), suggesting that targeting their crosstalk could provide new therapeutic strategies [114].

Understanding the molecular details of Hippo/Wnt crosstalk opens new avenues for innovative treatments in cancer and fibrosis, as YAP acts as a crucial intersection point and potential drug target [105, 107, 109, 110, 114].

#### 2.4.5 PI3K/Akt Pathway

The Hippo/YAP and PI3K/Akt pathways play crucial roles in regulating cell growth, proliferation, and survival. Dysregulation of these pathways is associated with several diseases, such as cancer and metabolic disorders. Recent studies have revealed considerable interactions between these pathways, which affect disease progression and identify potential new therapeutic targets.

YAP and TAZ, effectors of the Hippo pathway, can upregulate components of the PI3K/Akt pathway, including GAB2 and IRS2, which enhance PI3K/Akt signaling and promote cell proliferation and survival in cancers and metabolic diseases [94, 95, 97, 115].

In diabetic nephropathy, YAP activation (due to Hippo pathway inhibition) suppresses phosphatase and tensin homolog (PTEN), a negative regulator of PI3K/Akt, further activating the PI3K/Akt pathway. Consequently, PI3K/Akt activation can inhibit the Hippo pathway, creating a positive feedback loop that accelerates disease progression [97].

The Hippo pathway integrates mechanical, polarity, and nutritional cues with PI3K/Akt signals to regulate tissue growth. Insulin/IGF‐1 signaling through PI3K/Akt is essential for YAP nuclear localization and cell proliferation in both Drosophila and mammalian tissues [116].

Crosstalk between the Hippo/YAP and PI3K/Akt pathways is observed in endometrial, oral, liver, colon, and hepatocellular carcinomas. Nuclear YAP1 is linked to poor prognosis and increased tumor aggressiveness [94, 95, 96, 98, 99]. In the liver, the combined dysregulation of the Hippo and PI3K/Akt pathways accelerates nonalcoholic fatty liver disease (NAFLD) and tumorigenesis [95].

Inhibition of the Hippo pathway and subsequent activation of YAP promote mesangial cell proliferation through the PI3K/Akt pathway, thereby contributing to disease progression [97].

High nuclear YAP1 and TAZ levels correlate with poor differentiation, advanced stage, and worse survival in cancers [96]. Moreover, levels of pathway components (e.g., GAB2, IRS2, p‐AKT) may serve as biomarkers for disease progression and therapeutic response [94, 95].

#### 2.4.6 ROR1–HER3–Long Noncoding RNA (lncRNA) Axis

The interplay between the Hippo/YAP pathway and the ROR1–HER3–lncRNA axis has emerged as a key mechanism in cancer metastasis, particularly in the context of bone metastases from breast cancer. Recent research highlights how these signaling networks interact through specific molecular events, with lncRNAs playing a central role.

The orphan receptor tyrosine kinase ROR1 phosphorylates HER3 at a unique site (Tyr1307) upon neuregulin stimulation, independent of other ErbB family members. The lncRNA MAYA is recruited as part of a protein complex (LLGL2–MAYA–NSUN6) following HER3 phosphorylation. This complex methylates Hippo pathway kinase MST1 at Lys59, leading to its inactivation. Inactivation of MST1 results in the activation of YAP target genes, which are implicated in tumor progression and metastasis [78, 117].

In breast cancer, ROR1–HER3 signaling inactivates Hippo/MST1 through lncRNA‐mediated methylation, which activates YAP and promotes bone metastasis [78].

#### 2.4.7 YAP/Thrombospondin 1 (THBS1)/Focal Adhesion Kinase (FAK) Axis

Recent research has highlighted the interaction between Hippo/YAP signaling and the YAP/THBS1/FAK axis, uncovering new mechanisms by which YAP influences cancer cell behavior through focal adhesion and related pathways.

YAP, when upregulated, promotes cell proliferation and migration by inducing phosphorylation of FAK in a TEAD‐dependent manner in both gastric and breast cancer cells [79, 80].

FAK activity supports the nuclear aggregation of YAP, creating a positive feedback loop that sustains oncogenic signaling [79].

In breast cancer, YAP–TEAD directly activates the transcription of THBS1, which functions upstream of FAK. Silencing THBS1 reverses YAP‐induced FAK activation and decreases focal adhesion and invasiveness [80]. In gastric cancer, YAP–TEAD targets HMGB1, which also acts upstream of FAK; silencing HMGB1 reverses YAP‐driven FAK activation and cell proliferation [79].

The YAP/THBS1/FAK and YAP/HMGB1/FAK axes are associated with increased tumor cell proliferation, migration, adhesion, and invasiveness, contributing to poor prognosis in both gastric and breast cancers. Inhibiting YAP–TEAD interaction (e.g., with verteporfin) or targeting FAK or its upstream regulators (THBS1, HMGB1) disrupts this signaling, leading to reduced cancer cell aggressiveness [79, 80].

#### 2.4.8 TAZ/IL‐34 Axis

Recent research highlights the importance of crosstalk between Hippo/YAP signaling and the TAZ/IL‐34 axis, particularly in shaping the tumor immune microenvironment and influencing cancer therapy outcomes.

YAP and TAZ, regulated by the Hippo pathway, serve as transcriptional coactivators that move between the cytoplasm and nucleus, influencing gene expression in response to various signals, including mechanical cues and cell density [81, 82, 118].

In triple‐negative breast cancer (TNBC), Hippo–YAP/TAZ signaling enhances tumor‐associated macrophage (TAM) infiltration through the TAZ/IL‐34 axis, establishing an immunosuppressive environment that decreases the effectiveness of immune checkpoint therapies [84].

Inhibiting the TAZ/IL‐34 axis may enhance the efficacy of immunotherapies in TNBC. The broader inhibition of YAP/TAZ–TEAD activity is also being explored to counteract tumor progression and immune evasion [83, 84].

Hyperactivation of YAP/TAZ is linked to tumor growth, metastasis, and immune evasion. The TAZ/IL‐34 axis specifically promotes an immunosuppressive microenvironment by recruiting TAMs, which can impair antitumor immune responses [83, 84, 86]. YAP/TAZ interacts with multiple signaling pathways (e.g., Wnt, TGF‐β, GPCR), integrating diverse signals to regulate both tumor cell behavior and the immune landscape [77, 82, 85, 118].

## 3 Synthetic Compounds and Other Approaches Targeting the Hippo/YAP Pathway

Given the association between elevated YAP/TAZ levels and various cancers, some therapeutic strategies targeting the Hippo pathway have focused on suppressing the functions and roles of YAP and TAZ, either directly or indirectly. Various approaches have been explored, including drugs that activate MST and LATS, inhibit YAP/TAZ, disrupt the YAP/TAZ–TEAD interaction, or target the palmitoylation pocket of TEAD [119].

Pharmacological inhibition of YAP/TAZ, either through small molecules such as verteporfin, statins, or dasatinib, or via genetic approaches like CRISPR, represents a promising strategy for addressing Hippo pathway dysregulation in cancer. Simultaneously, restoring the expression of tumor suppressors like LATS1/2 or MST1/2 can re‐establish normal Hippo signaling, thereby counteracting oncogenic YAP/TAZ activity. For instance, verteporfin inhibits YAP activity in liver cancer, reducing tumor growth both in vitro and in vivo [120]. Statins, commonly prescribed as cholesterol‐lowering agents, inhibit YAP/TAZ activity by suppressing the mevalonate pathway, which is crucial for the nuclear translocation of YAP/TAZ. Consequently, statins have demonstrated potential in reducing YAP/TAZ activity in breast and colorectal cancers, where hyperactive YAP/TAZ signaling is often a driving factor [121]. Moreover, dasatinib, a tyrosine kinase inhibitor, has been shown to inhibit YAP activity and reduce tumor invasiveness in pancreatic cancer models [122]. Additionally, G‐protein‐coupled receptors (GPCRs), such as cysteinyl leukotriene receptor antagonists, have been found to reduce YAP/TAZ nuclear localization, thereby inhibiting their activity in various cancer cell types [21]. Genetic or pharmacological re‐expression of tumor suppressors within the Hippo pathway, including activating LATS1/2 kinases and MST1/2 (mammalian Ste20‐like kinases), has also been explored. The re‐expression of LATS1/2 and other upstream Hippo pathway components using viral vectors has been investigated in cancer models [123]. In TNBC, CRISPR‐mediated *YAP* knockout reduced tumor growth and invasiveness [124]. In cancers with low *LATS1* expression, CRISPR activation of *LATS1* restored Hippo pathway function and suppressed YAP/TAZ oncogenic activity, thereby inhibiting tumorigenesis [125].

In summary, these studies demonstrated encouraging findings in different cancer models. However, further research is crucial to reach their clinical application.

## 4 Natural Products Targeting the Hippo/YAP Pathway

**FIGURE 5:**
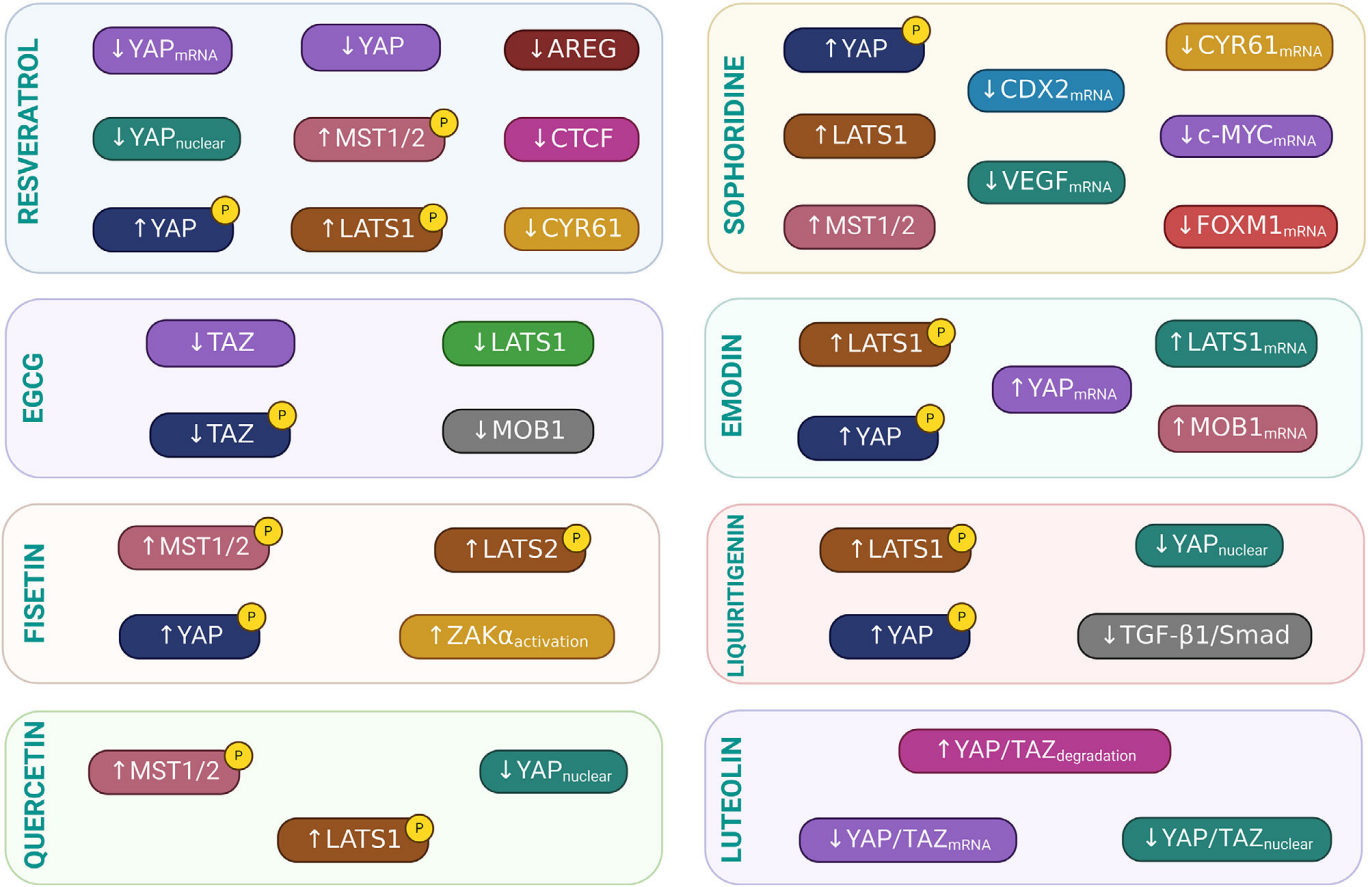
Effects of natural compounds in the Hippo pathway. The Hippo pathway is affected by natural agents that regulate the core Hippo kinases at transcriptional and posttranscriptional levels, resulting in the regulation of the expression of downstream genes. LATS1/2, large tumor suppressor kinase 1/2; MST1/2, mammalian sterile 20‐like 1/2; YAP, yes‐associated protein. *Source*: Created in https://BioRender.com.

For centuries, natural products derived from plants, marine organisms, and microorganisms have represented an invaluable source of abundant compounds with therapeutic activity against numerous diseases, including cancer. Recent research has identified several natural products that effectively target the Hippo/YAP pathway, offering new avenues for cancer therapy. The Hippo pathway is modulated by various natural compounds, which have been shown to influence key pathway components and downstream effects, including ursolic acid, *Pinus koraiensis*, liquiritigenin, decursin, and M*arsdenia tenacissima* extract [22] (Figure 5) (Table 2).

**TABLE 2:** Natural products that target the Hippo signaling pathway in different types of cancers.

Phytoconstituents	Mechanism of action	References
Resveratrol	In **human pancreatic cancer cells**—Suppress cell proliferation and induce apoptosis – Decrease expression of YAP at the mRNA and protein levels – Increase phosphorylation level of YAP–Decrease translocation of YAP to the nucleus In **human breast cancer cells**—Decrease the expression of YAP target genes, including *AREG*, *CTGF*, and *CYR61* – Inactivate RhoA, activating LATS1 and induction of YAP phosphorylation In **human thyroid FTC238 cells**—Increase expression of phosphorylated MST1/2, LATS1, and YAP – Decrease nuclear YAP and TAZ expression	[126, 127, 128]
Emodin	In **human HepG2 cells** – Induce the phosphorylation of LATS1 and YAP – Increase expression of *YAP*, *LATS1*, and *MOB1B* genes	[129]
Naringin	In **human endothelial cells**, it restores ox‐LDL‐induced YAP down‐regulation and apoptosis	[130]
Quercetin	Inhibit high glucose‐induced MC proliferation – Increase the phosphorylation level of MST1 and LATS1 – Reduce the expression of nuclear YAP and YAP–TEAD complex induced by high glucose levels	[131]
Luteolin	In **human and mouse TNBC cells**—Induce phosphorylation and degradation of YAP/TAZ – Reduce transcriptional activity and nuclear translocation of YAP/TAZ – Suppress the migration of TNBC cells	[132]
Epigallocatechin‐3‐gallat	In **mouse myoblast cells**—Increased myogenic differentiation – Reduce phosphorylation of TAZ and LATS1, not MST1/2–Increase translocation of TAZ to nucleus. In **tongue squamous cell carcinoma** – Decrease LATS1 and MOB1 protein levels – Reduce the total level of TAZ and phosphorylated TAZ	[133, 134]
Sophoridine	In **human lung cancer cells** – Suppress proliferation, colony formation, and migration of lung cancer cells – Increase expression of LATS1/2, MST1/2, and phosphorylated YAP – Downregulate the expression of some Hippo pathway target genes such as *CYR61*, *CDX2*, *FOXM1*, *c‐MYC*, and *VEGF*	[135]
Fisetin	In **human osteosarcoma cells** – Increase phosphorylation of MST1/2, LATS2, and YAP via ZAKα activation	[136]
Liquiritigenin	In **human HSC cells** – Increase phosphorylation of LATS1 and YAP – Prevent translocation of YAP to the nucleus – Suppress the expression of TGF‐β1/SMAD	[24]
Alantolactone	Increasing ROS‐induced LATS kinase activities and thus YAP1/TAZ phosphorylation	[137]

Abbreviations: FTC, follicular thyroid cancer cell; LATS1/2, large tumor suppressor kinase 1/2; MST1/2, mammalian sterile 20‐like 1/2; YAP, yes‐associated protein.

### 4.1 Ursolic Acid

Ursolic acid, found in different plants, inhibits cancer progression. At the structural level, ursolic acid is a pentacyclic triterpenoid with urs‐12‐ene‐28‐oic acid replaced by a beta‐hydroxy group at position 3. In MTT assays, it reduced the viability of gastric cancer cells and induced apoptosis in SNU484 and SNU638 cells in a dose‐dependent manner. Microarray analysis revealed that ursolic acid upregulates the Hippo pathway's upstream target gene, *RASSF1*, and downstream target genes. Additionally, *YAP1* and oncogene expression were significantly reduced. Furthermore, ursolic acid increased MST1, MST2, LATS1, RASSF1, and P‐YAP levels, whereas CTGF expression within gastric cells decreased. Therefore, ursolic acid demonstrates potential as a chemopreventive and chemotherapeutic agent by regulating the Hippo pathway through RASSF1, thereby reducing tumorigenesis, proliferation, and metastasis [138].

### 4.2 Pinecone

Pinecone, a traditional herbal product from China, contains 41 compounds, such as alpha‐pinene, limonene, and beta‐pinene. On the basis of RNA sequencing, it exhibits antitumor activity in MGC‐803 cells, with approximately 100 upregulated genes and 57 downregulated genes [139]. Thiosemicarbazones, associated with limonene, significantly inhibit a broad range of cancer cell lines, including prostate cells. Its subtypes 5, 6, 8, 9, and 10 exerted a potent anticancer effect. Notably, the 4‐fluorobenzaldehyde derivative 10 was the most lethal for prostate cancer cells [140].

### 4.3 Liquiritigenin

Liquiritigenin is a dihydroxyflavonone derived from the roots of *Glycyrrhiza uralensis* with two hydroxy substituents at different positions. 5‐Deoxyflavonone is present in a solid form and contains a flavane‐3‐one moiety evaluated with 2 phenyl 3,4 dihydro 2*H*‐1‐benzopyran with a ketone at the carbon C3. Liquiritigenin, acting on the Hippo pathway, effectively inhibits liver fibrosis. It reduces the degeneration area in the liver, inflammatory cell infiltration, and alpha‐smooth muscle actin staining in mice. Additionally, it blocks TGF‐β1‐promoted phosphorylation in SMAD3 and regulates transcript levels of plasminogen activator inhibitor 1 and matrix metalloproteinase 2 in LX‐2 cells. Liquiritigenin also activates large tumor suppressor kinase‐1 and initiates YAP phosphorylation, inhibiting the expression of TGF‐β1/SMAD signaling agents [24].

### 4.4 Decursin

Decursin is another potential anticancer natural agent that acts through the Hippo pathway. In a study, decursin suppressed HepG2 liver cancer cells, leading to significant cell proliferation arrest, cell cycle disruption, and apoptosis promotion in a dose‐dependent manner. Decursin achieves these effects by regulating phosphorylated LATS1 and beta‐TRCP, which degrade YAP. Furthermore, the apoptotic effect can be reversed using a selective MST1/2 inhibitor, XMU‐MP‐1, confirming the role of Hippo–YAP signaling [25].

### 4.5 Extracts From *Marsdenia tenacissima*

M. tenacissima extract exerts its overall anticancer effect through its five main C21 steroid fractions [141]. One of these fractions, FR5, induces apoptosis in HepG2 and Bel7402 cells. Additionally, it inhibits the Hippo pathway, leading to YAP inactivation and PTEN elevation. Increased PTEN levels inhibit the PI3K/AKT signaling pathway, preventing cell spread. In addition, FR5 can reduce tumor growth in xenograft mouse models with hepatocellular carcinoma by coordinating the inhibition of YAP in the Hippo pathway, demonstrating FR5 as a potential therapeutic option for this cancer. In another study, matrine exerted its anticancer effect through the LATS2‐Hippo axis. It inhibited SW480 cell survival by stimulating MIEF1‐related mitochondrial division via the LATS2‐Hippo pathway [27].

### 4.6 Curcumin

Curcumin is a beta‐diketone where feruloyl groups substitute two hydrogens; it modulates the Hippo pathway in a dose‐dependent manner. Curcumin downregulates YAP and TAZ, which competitively interfere with WW domain‐containing E3 ubiquitin protein ligase 1 (WWP1) binding to the Krüppel‐like factor 5 (KLF5), decreasing KLF5 protein stability. Additionally, downstream targets of YAP and TAZ, including integrin beta 2, cyclin‐dependent kinase 6, AXL receptor tyrosine kinase, and Cyr61, are downregulated by curcumin [142].

### 4.7 Resveratrol

Resveratrol is a polyphenolic phytoalexin derived from stilbene; it is generated in plants with the aid of an enzyme called stilbene synthase. Under ultraviolet radiation, the *trans* version of this compound may change to the *cis* form. Resveratrol suppresses the growth of cancer cells via the Hippo pathway. It inhibits anaplastic thyroid carcinoma in murine models. Exposure of follicular thyroid cancer cells (FTC133 and FTC238 cell lines) to resveratrol reduced their viability in a dose‐dependent manner compared to control, as demonstrated by colony‐forming assays. Likewise, resveratrol significantly diminished the tumorigenesis of FTC238 cancer cells in in vivo assays. Moreover, western blot analysis confirmed its impact on the Hippo signaling pathway in FTC cells, possibly linked to decreased ST6GAL2 expression [128].

### 4.8 Epigallocatechin‐3‐Gallate (EGCG)

On the other hand, EGCG is a gallate ester formed through the condensation of gallic acid with the (3*R*)‐hydroxy group of (−) epigallocatechin. A study demonstrated that EGCG reduced total TAZ and p‐TAZ protein levels in a tongue squamous cell carcinoma (CAL27 cells) cell line. The treatment also significantly decreased the levels of LATS1 and MOB1 proteins. Furthermore, in experiments conducted on SCC15 cells, EGCG exhibited a similar effect to that observed in CAL27 cells, thus revealing the role of the Hippo‐TAZ signaling pathway [134].

In conclusion, numerous studies have demonstrated that several natural products can target and modulate the Hippo/YAP pathway by influencing key components, including YAP, MST1, MOB1, and LATS1. Therefore, these compounds have the potential to be used against cancer; however, extensive analysis is needed before they can be approved.

### 4.9 Mechanisms of Drug Resistance via Hippo/YAP Pathway

Targeting the Hippo/YAP pathway is emerging as a promising strategy to overcome drug resistance in various types of cancer. Inhibiting YAP, a key effector of the Hippo pathway, can resensitize resistant cancer cells to therapies and enhance antitumor immune responses. YAP is often upregulated in drug‐resistant cancer cells, promoting cell survival, proliferation, and migration, which contributes to resistance against targeted therapies (e.g., trastuzumab, RAF/MEK inhibitors, temozolomide) and cytotoxic drugs [143, 144, 145, 146, 147, 148, 149, 150]. YAP enhances the expression of immune suppression markers (e.g., PD‐L1), leading to decreased T cell infiltration and reduced antitumor immunity [143, 144].

In some cancers, the loss of tumor suppressors (e.g., STK11) increases dependence on YAP, allowing cells to bypass the effects of other targeted therapies [148, 151]. Inhibiting YAP (using siRNA or verteporfin) in trastuzumab‐resistant cells reduces tumor growth, decreases immune suppression markers, and enhances T cell activity, both in vitro and in vivo [143, 144]. YAP is overexpressed in temozolomide‐resistant cells. Inhibiting YAP restores sensitivity to temozolomide and reduces tumor size in animal models [145]. Combined inhibition of YAP and other pathways (e.g., RAS‐MAPK, BET, MAP3K3) enhances sensitivity to targeted therapies and induces cancer cell death [146, 148, 151].

Disrupting Hippo/YAP signaling enhances the sensitivity of different cancer cell types to cytotoxic drugs by altering drug transporter expression and metabolism [149, 150, 152]. Agents such as verteporfin and MAP3K3 inhibitors demonstrate preclinical efficacy in overcoming resistance [143, 144, 145, 146]. Targeting YAP alongside standard treatments enhances the anti‐cancer effects and may help prevent or delay resistance [145, 146, 148, 151].

The following Table 3 outlines the evidence for overcoming resistance.

**TABLE 3:** Mechanisms of drug resistance via Hippo/yes‐associated protein (YAP) pathway.

Resistance targeted	Cancer type	YAP inhibition effect	Refs.
Trastuzumab	HER2+ (breast/gastric)	Restores drug sensitivity, boosts immunity	[143, 144]
Temozolomide	Glioblastoma	Resensitizes cells, shrinks tumors	[145]
RAF/MEK, CDK4/6, BET	Lung/Breast	Induces cell death, overcomes resistance	[146, 148, 151]
Cytotoxic drugs	Multiple cancers	Increases drug effectiveness	[149, 150, 152]

### 4.10 Effect of Hippo/YAP Pathway in Combination Strategies

Targeting the Hippo/YAP pathway is an emerging strategy in cancer therapy, particularly when combined with other treatments. Combination approaches that inhibit YAP/TAZ, often in conjunction with other pathways, can overcome drug resistance, suppress tumor growth, and enhance the effectiveness of existing therapies.

YAP and TAZ are crucial effectors of the Hippo pathway, driving tumor growth, metastasis, and drug resistance when hyperactivated. Their activity is associated with immune suppression and abnormal blood vessel formation in tumors [83, 153, 154, 155].

Combining metabolic stress (e.g., glucose restriction) and metformin with Hippo pathway activation selectively kills cancer cells by inhibiting YAP/TAZ, independent of other common metabolic pathways [156].

YAP activation provides a parallel survival pathway in cancers treated with RAF‐ and MEK‐targeted therapies, resulting in resistance. Inhibiting YAP alongside these therapies produces synthetic lethality and enhances treatment response [148, 151, 153].

High YAP/TAZ activity predicts a poor response to targeted therapies, making it a valuable biomarker for selecting therapies [148, 153, 155].

Several small molecules and nanomedicines are being developed to inhibit YAP/TAZ–TEAD interactions, with promising preclinical results [119, 155, 157].

Combining Hippo/YAP‐targeted agents with other treatments (e.g., kinase inhibitors, immunotherapy, radiotherapy) shows synergistic effects and may help prevent or overcome drug resistance [83, 148, 151, 153, 155, 157].

The following Table 4 summarizes the combination strategies and effects of the Hippo/YAP‐targeting agents.

**TABLE 4:** Combination strategies of the Hippo/yes‐associated protein (YAP)‐targeting agents.

Combination approach	Cancer type/model	Effect/Outcome	Refs.
BET + RAS‐MAPK inhibitors + YAP/TEAD inhibition	NSCLC (KRAS/BRAF mutant)	Overcomes resistance, induces tumor regression	[148, 151, 153]
YAP/TAZ–TEAD inhibitors + chemo/immunotherapy	Various	Enhances immune response, suppresses tumor growth	[83, 155]
Metformin + glucose restriction (Hippo activation)	General cancer models	Induces cancer cell death via YAP/TAZ inhibition	[156]
Hippo pathway‐activating nanomedicine + radiotherapy	Triple‐negative breast	Inhibits growth/metastasis, increases radiosensitivity	[157]
Src inhibitors + YAP targeting	NSCLC	Potential to overcome resistance, under investigation	[74]

Abbreviation: NSCLC, non‐small‐cell lung cancer.

## 5 Preclinical and Clinical Studies of Compounds Targeting the Hippo/YAP Pathway in Cancer

Several preclinical studies have provided valuable insights into the anticancer effects of natural products targeting the Hippo/YAP pathway. However, these studies must be validated through upcoming clinical trials for the management of cancer patients [158, 159]. As previously discussed, the Hippo pathway is associated with various signaling cascades that regulate organ size, tissue homeostasis, and cell proliferation [13]. The critical elements of this pathway are the kinases MST1/2 and LATS1/2 and the downstream effectors YAP and TAZ [160]. Recent evidence suggests that the Hippo pathway regulates metabolic reprogramming to facilitate the progression of malignant tumors [161, 162]. The Hippo–YAP pathway link of glucose metabolism is essential to promoting tumor progression. The dysregulation of the Hippo pathway results in the aberrant activation of the transcriptional co‐activators YAP and TAZ in various types of cancer, contributing to oncogenesis. YAP/TAZ interacts with transcription factors such as TEADs in the nucleus to promote gene expression related to cell proliferation, survival, and metastasis [121, 123]. Therefore, the Hippo/YAP signaling pathway is a promising therapeutic target in cancer due to its critical role in tumor initiation, progression, and metastasis.

Conventional cancer therapies often exhibit limited efficacy and significant side effects. Natural products derived from plants, animals, and microorganisms have long been a rich source of therapeutic agents in cancer treatment. Their diverse chemical structures and biological activities make them attractive candidates for drug discovery and development. Eventually, patients susceptible to dysregulation of the Hippo/YAP signaling pathway could be treated with a personalized medicine approach, allowing for a specific treatment tailored to the type of impairment. Personalized medicine for Hippo/YAP signaling pathway dysregulation in cancer could initially consist of adjuvant treatments with natural products that allow for a reduction in the dosage of traditional anticancer drugs. However, genetic predisposition studies could involve identifying the dysregulation of the Hippo/YAP signaling pathway and applying different natural products as a form of prevention. Below is a comprehensive overview of preclinical and clinical studies investigating the therapeutic potential of diverse compounds targeting the Hippo/YAP pathway in cancer.

### 5.1 Preclinical Studies Involving Hippo/YAP in Cancer Therapy

Within the efforts to demonstrate that certain natural compounds have antitumor properties, and their biological effects are exerted through the hypo pathway, preclinical study models (in vivo models) represent a great alternative since they are also supported in demonstrations made in in vitro models (different cell lines for various types of cancer), where the mechanism of action of diverse natural compounds has been elucidated. In concordance with the above, scutellarin treatment in mice increased pYAP levels and lowered YAP1 expression, which was directly associated with tumor growth inhibition and was supported by cell line studies in the same research experiment [163]. Likewise, a research group demonstrated that EGCG suppressed YAP activation in TNBC cells [164]. Mechanistically, EGCG inhibited the nuclear translocation of YAP, leading to the downregulation of YAP target genes involved in cell proliferation and migration. Similarly, Lin et al. [165] demonstrated that gambogic acid inhibits YAP/TAZ function and suppresses gastric cancer growth in preclinical models. Moreover, gambogic acid exhibited synergistic effects with chemotherapy drugs, suggesting its potential as an adjuvant therapy for gastric cancer. On the basis of the above experimental evidence, it is concluded that treatment with gambogic acid and EGCG inhibits YAP/TAZ‐mediated transcriptional activity and downregulates YAP/TAZ target genes associated with cell proliferation and survival.

On the other hand, a research group using a lung cancer xenograft model demonstrated that the aqueous extract of *Taxus chinensis* var. *mairei* induces apoptosis of NSCLC cells, activating the ATF3–Hippo–YAP pathway in vivo, through a reduction in tumor volume and weight in nude mice, and that this was a result of upregulation of ATF3, p‐MOB1, and p‐YAP (Ser397) [166]. Similarly, Jin et al. [167], using a similar model of lung cancer, demonstrated in a preclinical study that the acetate/ethyl extract of *Celastrus orbiculatus* (COE) substantially inhibited the activity of non‐small cell lung cancer in vitro and in vivo by inhibiting proliferation, arresting the cell cycle, and promoting apoptosis. COE strongly activated Hippo signaling and inhibited YAP expression and nuclear retention. COE‐induced activation of Hippo signaling was associated with ROS‐mediated phosphorylation of MOB1 [167].

Within the same context, Sha et al. investigated the effects of curcumin on YAP/TAZ activity in pancreatic cancer cells. They found that curcumin inhibits YAP/TAZ activation and suppresses pancreatic cancer progression by inhibiting the expression of YAP/TAZ target genes involved in cell proliferation and metastasis [168]. Recently in 2021, Wang et al. demonstrated that ginsenoside Rh2 inhibits YAP‐mediated drug resistance in ovarian cancer cells. Treatment with ginsenoside Rh2 sensitized ovarian cancer cells to chemotherapy drugs by inhibiting YAP‐mediated expression of drug‐resistance genes [169]. In their review article, Kumar et al. highlighted various preclinical and clinical studies on various herbal sources and phytochemicals (ailanthone, apigenin, catechol, curcumin, emetine, pterostilbene, shikonin, and wogonin) with anticancer properties that target the Hippo pathway [159]. These findings highlight the potential of natural products to enhance chemotherapy efficacy in ovarian cancer by targeting the Hippo/YAP pathway, paving the way for more effective combination therapies.

Regarding the potential treatment of gynecological cancers through the modulation of the Hippo/YAP pathway, a study carried out in an in vivo breast cancer model (a xenograft breast cancer model) showed that phosphorylated YAP1/TAZ proteins were subject to nuclear exclusion and proteasomal degradation, such that the growth of ALT‐treated tumor cells was inhibited in both breast cancer study models, in vitro and in vivo, suggesting that alantolactone can be used to target the ROS‐YAP pathway driving tumor cell proliferation and so could be a potent antitumoral agent [137]. Finally, innovative approaches, such as supramolecular nanomedicine, are being developed to activate the Hippo pathway in TNBC. These strategies aim to inhibit tumor growth and metastasis by converting inactive nanospheres into active nanofibers that modulate the Hippo pathway [170]. Table 5 summarizes preclinical and clinical trials investigating the anticancer role of natural constituents via the Hippo pathway.

**TABLE 5:** Preclinical and clinical trials of natural constituents illustrating their anticancer role by targeting the Hippo pathway.

Study title	Type	Natural product	Cancer type	Methodology	Findings/Results	Reference
“EGCG Suppresses YAP Activation in Triple‐Negative Breast Cancer Cells”	Preclinical	EGCG (epigallocatechin gallate)	Triple‐negative breast cancer	Female CB‐17 severe combined immunodeficient (SCID) mice (6–8 weeks old) received 100 mg/kg of EGCG dissolved in 100 µL of water every 2 days via oral gavage, whereas the mock‐treated group received only water. Mice were sacrificed when the mock group's tumor volume reached approximately 1000 mm^3^. Tumors were excised, sectioned, and stained with Ki67 or PARP antibody. Relative ratio of TUNEL‐positive cells was calculated from 10 randomly selected microscopic fields in each group	Inhibition of YAP activation and cell proliferation	[164]
“Gambogic Acid Inhibits YAP/TAZ Function and Suppresses Gastric Cancer Growth”	Preclinical	Gambogic acid	Gastric cancer	Various assays, including cell colony formation, MTT, transwell, and flow cytometry, were conducted to investigate the functional roles of circ_ASAP2, miR‐33a‐5p, and CDK7 on gambogic acid (GA)‐induced gastric cancer progression in cell lines AGS and HGC‐27, along with the normal human gastric epithelial cell line GES‐1 For in vivo studies, gastric cancer cells were injected into 6‐week‐old nude mice. GA was injected intraperitoneally every 3 days starting 10 days post‐injection. After 22 days, the mice were euthanized for tumor excision. Tumor growth curves were generated, and tumor weights were recorded	Inhibition of YAP/TAZ function and tumor growth suppression	[165]
“The Anticancer Effects of Ginsenoside Rh2 on YAP‐Mediated Drug Resistance in Ovarian Cancer”	Preclinical	Ginsenoside Rh2	Ovarian cancer	Ovarian cancer cell lines treated with of Ginsenoside Rh2 were used to assess cell viability and to perform several assays	Inhibition of YAP‐mediated drug resistance and sensitization to chemotherapy	[169]
“Curcumin Inhibits YAP/TAZ Activity and Suppresses Pancreatic Cancer Progression”	Preclinical	Curcumin	Pancreatic cancer	HT29 and SW480 cells were treated with curcumin and/or doxycycline (DOX), and their cell viability, colony‐forming ability, migration, and invasion were assessed. Additionally, genes and proteins related to yes‐associated protein 1 (YAP) and PDZ‐binding motif (TAZ) signaling were analyzed via reverse transcription quantitative real‐time PCR (RT‐qPCR), western blotting, and immunofluorescence. A nude mouse xenograft tumor model was then established, and YAP and Ki67 expression levels were evaluated using immunohistochemistry (IHC) staining	Inhibition of YAP/TAZ activity and tumor growth inhibition	[171]
Aqueous extract of *Taxus chinensis* var. *mairei* regulates the Hippo–YAP pathway and promotes apoptosis of non‐small cell lung cancer via ATF3 in vivo and in vitro	Preclinical	Aqueous extract of *Taxus chinensis* var. *mairei* (AETC)	Non‐small‐cell lung cancer	Xenograft model (BALB/c Nude Mouse inoculated with NCI‐1975 cells)	Reduced the tumor volume and weight in nude mice. Upregulation of ATF3, p‐MOB1, and p‐YAP (Ser397)	[166]
The ethyl acetate extract from *Celastrus orbiculatus* suppresses non‐small‐cell lung cancer by activating Hippo signaling and inhibiting YAP nuclear translocation	Preclinical	Ethyl acetate extract from *Celastrus orbiculatus* (COE)	Non‐small‐cell lung cancer	Xenograft model (BALB/c Nude Mouse inoculated with H1299 cells)	COE strongly activated Hippo signaling and inhibited YAP expression and nuclear retention. Activation of Hippo signaling induced by COE was associated with ROS‐mediated phosphorylation of MOB1	[167]
Allosteric Inhibitors of SHP2 with Therapeutic Potential for Cancer Treatment	Preclinical	Allosteric SHP2 inhibitor (23)	Acute monocytic leukemia	Xenograft model (BALB/c Nude Mouse inoculated with MV4;11 cells) MV4;11	Suppresses MAPK signaling pathway and YAP transcriptional activity and shows tumor growth inhibition	[172]
Alantolactone is a natural product that potently inhibits YAP1/TAZ through promotion of reactive oxygen species accumulation	Preclinical	Alantolactone	Breast cancer	Xenograft model (BALB/c Nude Mouse inoculated with MDA‐MB‐231cells)	Alantolactone can be used to target the ROS‐YAP pathway driving tumor cell growth and so could be a potent anticancer drug	[137]

Abbreviations: ATF3, activating transcription factor 3; MOB1, monopolar spindle one binder 1; SHP2, nonreceptor protein tyrosine phosphatase; YAP, yes‐associated protein.

### 5.2 Current Clinical Trials Involving Hippo/YAP in Cancer Therapy

Preclinical data become the basis for clinical studies that validate natural products targeting the Hippo/YAP Pathway. Thus, several researchers have conducted clinical trials exploring cancer management using natural products, focusing on this pathway. For instance, Hsiang et al. recently conducted a phase I clinical trial to evaluate the safety and efficacy of CA‐170, an amino acid‐inspired small molecule, in patients with advanced solid tumors. Preliminary evidence indicates that CA‐170 possesses antitumor activity by inhibiting YAP/TAZ and TEAD transcription factors, and it was well‐tolerated and had manageable adverse effects [171].

Resveratrol has been reported to modulate the Hippo/YAP pathway in preclinical studies. A phase II clinical trial by Gilgenkrantz et al. investigated resveratrol's efficacy in patients with colorectal cancer. Although the primary endpoint of progression‐free survival was not met, resveratrol treatment was associated with improvements in secondary endpoints, including overall survival and quality of life. These findings suggest that resveratrol may have potential as an adjuvant therapy for colorectal cancer patients, although further studies are needed to confirm its efficacy [110].

As mentioned above, in preclinical studies, the administration of EGCG has shown encouraging results for cancer management by targeting the Hippo pathway. The results were well supported by clinical evidence. Jatoi et al. evaluated the safety and efficacy of green tea extract, rich in polyphenols such as EGCG, in patients with prostate cancer undergoing active surveillance [173]. The study demonstrated that green tea extract was well‐tolerated, with no dose‐limiting toxicities observed. Although no significant changes in prostate‐specific antigen levels were observed, the treatment was associated with favorable changes in biomarkers related to prostate cancer progression.

On the other hand, transcriptional co‐regulators YAP and TAZ, which interact with the TEAD family of transcription factors, play a significant role in cancer development and progression. They contribute to oncogenic traits such as sustained proliferation, inhibition of apoptosis, and resistance to therapies, making them attractive targets for cancer therapy. Several drugs that inhibit YAP/TAZ activities are already in clinical use, and new inhibitors are under development. Since 2018, significant progress has been made in developing small‐molecule inhibitors targeting the YAP/TAZ–TEAD interaction. These inhibitors are classified into external TEAD ligands, non‐covalent TEAD ligands targeting the palmitate pocket, and covalent TEAD ligands. The first molecules to enter clinical trials are non‐covalent TEAD ligands, underscoring the therapeutic potential of this pathway for cancer treatment [174]. In this regard, a first‐in‐human Phase 1 trial of VT3989, a YAP/TEAD inhibitor, is currently underway. This trial focuses on patients with advanced solid tumors, particularly those with malignant mesothelioma or neurofibromatosis 2 (NF2) mutations. VT3989 has demonstrated promising preclinical activity by blocking YAP function by inhibiting TEAD palmitoylation. The trial aims to assess the safety, tolerability, and recommended Phase 2 dose, with early results indicating that VT3989 is safe, well‐tolerated, and capable of producing durable antitumor responses [175].

The development of medications from natural products targeting the Hippo/YAP pathway destined for cancer treatment is challenging. The pharmacokinetics and bioavailability of natural products must be standardized to enhance their efficacy and safety in clinical settings. Most natural products targeting the Hippo/YAP pathway have low solubility in aqueous media, which will describe challenging pharmacokinetics. If a molecule has low solubility, its possibility of reaching the bloodstream is reduced, even more so when a description of low permeability is added. Therefore, the pharmacological effect can be reduced, aberrant, or variable. Most preclinical and clinical studies do not provide further information about the purity of the compounds, which is crucial when dealing with molecules isolated from natural sources. Intrinsically, some natural compounds exhibit instability, which can contribute to low bioavailability, potentially compensated by an increase in the dose, but with a risk of increased adverse effects. In nature, natural products targeting the Hippo/YAP pathway require a pharmaceutical form that facilitates their dosage and administration. Even more so in the case of cancer, a controlled and vectorizable release system is highly desired. Moreover, developing combination therapies that target multiple signaling pathways may represent a promising strategy to overcome drug resistance and improve treatment outcomes in cancer patients. Ultimately, considerable effort should be devoted to identifying predictive biomarkers in patients to identify subpopulations of cancer patients most likely to benefit from treatment with natural products.

In summary, preclinical and clinical research findings provide encouraging evidence to continue clinical trials that allow using natural products targeting the Hippo pathway as safe and effective anticancer adjuvants and/or therapies.

### 5.3 Limitations of Hippo/YAP‐Targeting Drugs

Targeting the Hippo/YAP pathway represents a promising strategy for cancer therapy, as YAP is a crucial driver of tumor growth and drug resistance. However, drugs that target the Hippo/YAP pathway face significant limitations, including the development of resistance, the complexity of the pathway, and difficulties in identifying effective drug combinations and biomarkers.

Cancer cells can activate parallel survival pathways, such as the RAS‐MAPK pathway, to bypass Hippo/YAP inhibition, resulting in drug resistance and decreased effectiveness of single‐agent therapies [148, 151, 176, 177].

Co‐occurring mutations (e.g., STK11 loss) can create dependencies on YAP/TEAD, resulting in some tumors being resistant to Hippo/YAP‐targeted drugs unless combined with other pathway inhibitors [151, 153, 176].

YAP activation can happen independently of the canonical Hippo pathway, complicating predictions of drug response and requiring combination strategies for effective treatment [153, 176, 177]. Many Hippo/YAP‐targeting drugs demonstrate limited effectiveness as monotherapies because of the rapid development of resistance and tumor adaptation [153, 177, 178]. There is a need for robust biomarkers to identify patients who will benefit from Hippo/YAP‐targeted therapies and to monitor treatment responses [178, 179]. The role of YAP can differ among cancer types and even within subtypes (e.g., YAP may act as a tumor suppressor in some breast cancers), complicating the design of universal therapies [178, 179, 180, 181].

The following Table 6 summarizes the limitations and strategies of Hippo/YAP‐target drugs.

**TABLE 6:** Limitations and current strategies associated with Hippo/yes‐associated protein (YAP)‐target drugs.

Limitation	Potential solution/approach	Refs.
Resistance via parallel pathways	Combination therapies (e.g., YAP + MEK)	[148, 151, 153, 176]
Tumor heterogeneity	Personalized/Biomarker‐driven approaches	[151, 179, 180, 181]
Pathway complexity	Targeting multiple nodes in the pathway	[153, 176, 178, 182]

## 6 Conclusion and Future Directions

*Key findings*: The Hippo/YAP pathway is of interest in the current panorama of cancer research and therapy because the cellular characteristics that maintain the conservation of the junction between cells depend largely on it. This pathway is a critical regulator of cell growth, tissue homeostasis, and organ size; thus, its dysregulation is associated with various diseases, including cancer. It is widely considered that this pathway is related to the control of tumor phenotypes and characteristics of cancer, which confer capabilities ranging from initiation to the progression of this pathology. Thus, it is necessary to insist on more significant knowledge of this pathway. Preclinical and clinical studies have suggested the potential of several synthetic compounds and natural products that target the Hippo/YAP pathway as therapeutic agents for combating cancer. *Challenges*: However, extensive research is needed to optimize their therapeutic efficacy and safety for clinical applications. For example, natural products exhibit varied chemical structures, pharmacokinetics, and bioavailability. In addition to the fact that few characterized compounds can modulate it, another challenge is that these compounds exhibit low selectivity for modulating this pathway. *Future directions*: Using compounds targeting the Hippo/YAP pathway as medication will require rigorous standardization of dosing, formulations, and administration routes. It will also be crucial to thoroughly analyze the possible interactions of these compounds with drug transporters, metabolizing enzymes, and cellular uptake mechanisms. On the other hand, it is well‐known that current cancer therapies exhibit limitations because of drug resistance. Regarding this, combining natural products with conventional therapies would enable the development of novel treatment regimens and enhance clinical outcomes. In this context, clinical trials are crucial for establishing the therapeutic efficacy of these compounds against cancer. In summary, natural products targeting the Hippo/YAP pathway hold promising therapeutic potential for cancer; however, interdisciplinary research and personalized approaches are essential to fully realize their benefits.

## Author Contributions

Gerardo Leyva‐Gómez, Javad Sharifi‐Rad, and William C. Cho conceptualized the idea and structured and oversaw the article. Rajni Bala, Reecha Madaan, Onkar Bedi, Amrinder Singh, Ayushi Taneja, and Renu Dwivedi conducted literature reviews and wrote the first draft of the manuscript. Gabriela Figueroa‐González, Octavio Daniel Reyes‐Hernández, Laura Itzel Quintas‐Granados, Dietrich Büsselberg, and Hernán Cortés contributed to the writing, compiled information, made the figures, and performed edition and revision of the manuscript. All authors read and approved the final version of the manuscript.

## Ethics Statement

The authors have nothing to report.

## Conflicts of Interest

The authors declare no conflicts of interest.

## Data Availability

The authors have nothing to report.
